# Trauma care performance and patient outcomes - insights from a major trauma centre in Sweden

**DOI:** 10.1007/s00068-026-03364-5

**Published:** 2026-10-27

**Authors:** Jesper Eriksson, Emma Larsson, Bjarni Arnason, Daniel Ohlén, Magnus Hedberg, Malin Jonsson Fagerlund, Erik von Oelreich

**Affiliations:** 1 Perioperative Medicine and Intensive Care, Karolinska University Hospital, Solna, Stockholm, SE-171 76 Sweden; 2 Section for Anesthesiology and Intensive Care Medicine, Department of Physiology and Pharmacology, Karolinska Institutet, Stockholm, Sweden

**Keywords:** Trauma, Multiple trauma, Outcome, Injury severity, Computed tomography, Mortality

## Abstract

**Purpose:**

Trauma is a leading cause of death among individuals under 45 years of age. Evaluating trauma system performance over time is important but inherently difficult due to concurrent changes in patient demographics, case-mix and care processes. The aim of this study was to describe temporal changes in patient demographics, injury severity and trauma care at a major trauma centre in Sweden from 2017 to 2023. We hypothesised that risk-adjusted 30-day mortality decreased and that time to computed tomography was reduced over the study period.

**Methods:**

A retrospective cohort study was conducted using data from the Karolinska Trauma Registry, including all trauma patients aged > 15 years fulfilling criteria for full trauma team activation, admitted between January 2017 and August 2023. Multivariable logistic regression was used to assess changes in 30-day mortality over time, adjusted for age, sex, injury severity category, ASA score and injury type. Time from arrival to computed tomography was analysed by linear regression and, because the distribution was skewed, by quantile (median) regression.

**Results:**

A total of 5,615 trauma patients were included. Over the study period, the patient population became older, and ASA scores increased, while injury severity and the proportion of penetrating injury remained unchanged. In multivariable logistic regression with 2017 as reference, the odds of 30-day mortality in 2022–2023 were lower (OR 0.61, 95% CI 0.43–0.88, *P* = 0.008) after adjusting for confounders. Median time to computed tomography decreased from 31 to 21 min; the adjusted difference in 2022–2023 compared with 2017 was − 7.5 min by linear regression (*P* = 0.014) and − 10.4 min by quantile regression (95% CI − 11.5 to − 9.2, *P* < 0.001).

**Conclusion:**

Our findings are consistent with improvements in trauma care within a mature trauma system, with shorter median time to computed tomography and lower 30-day mortality despite an aging population with increasing comorbidity. However, the specific drivers of these changes are difficult to identify, and future studies should explore how individual components of pre-hospital and in-hospital care contribute to trauma outcomes.

**Supplementary Information:**

The online version contains supplementary material available at https://doi.org/10.1007/s00068-026-03364-5.

## Background

Evaluating the effectiveness of trauma systems over time is inherently complex [1]. Although improvements in patient outcomes and survival may be expected as trauma systems mature, demonstrating such progress quantitatively remains challenging. An important confounding factor is the aging population, as the proportion of older trauma patients continues to rise [2]. This trend is expected to persist, and older adults often present with greater comorbidity burden and more complex injuries, influencing outcomes [3]. Moreover, temporal changes in case mix – including variations in injury patterns, injury severity, and pre-existing health conditions – further complicate interpretation. Such evolving patient characteristics risk obscuring genuine advances in system performance, as observed outcomes may reflect population-level changes rather than improvements in trauma care itself. Understanding how trauma care performance changes over time is therefore essential for identifying opportunities to optimise resource use in the context of an increasingly older and more complex trauma population.

Advancements in trauma care, encompassing both pre-hospital and in-hospital factors, may improve outcomes through earlier interventions and improved system efficiency [4]. Faster access to definitive care, improved diagnostic efficiency - including shorter time to computed tomography (CT) - and advances in surgical management may all contribute to improved survival [5]. However, the relative contribution of these factors is difficult to quantify and account for in longitudinal studies of trauma care.

The aim of this study was to describe temporal changes in patient demographics, injury severity, and trauma care at a major trauma centre over a period of six years and eight months. We hypothesised that risk-adjusted 30-day mortality decreased and that time from hospital arrival to computed tomography was reduced during the study period.

## Methods

### Study design

This observational cohort study used data from the Karolinska Trauma Registry and included all trauma patients aged > 15 years with fulfilling criteria for full trauma team activation, admitted from January 1st 2017 to August 30th 2023. At the time of data acquisition, August 30th 2023 was the most recent date with complete registry entries, thereby defining the study period. Because case capture for 2023 was incomplete, analyses restricted to that year risked being underpowered; consequently, data from 2022 to 2023 were pooled for year-specific analyses. To assess the potential impact of seasonal imbalance resulting from pooling, two sensitivity analyses were performed: one entering 2022 and 2023 as separate categories, and another restricting all study years to January-August to ensure identical calendar-month coverage.

The study was approved by the Swedish Ethical Review Authority (Dnr 2023-03869-01) with informed consent waived and reported in adherence to the STROBE guidelines [6].

The Karolinska Trauma Registry includes patients admitted through the trauma unit following trauma team activation. Patients with burn injuries < 18% without inhalation injury, chronic subdural hematomas, asphyxia without traumatic injuries, drowning, or hypothermia without traumatic injuries are excluded. Injury coding and ISS calculation are performed by dedicated registry nurses trained and certified in Abbreviated Injury Scale (AIS) coding.

A two-tiered trauma alert system is used. Full trauma team activation is triggered by physiological abnormalities or specific anatomical injuries, whereas a limited trauma team activation is based solely on mechanism of injury [7, 8]. For this study, only patients admitted following full trauma team activation were included. Injury severity was assessed using ISS derived from AIS 2005 edition (updated 2008). An injury to a specific body region was classified as serious when AIS > 2.

### Study setting

This research was conducted at the Karolinska University Hospital in Stockholm, the largest trauma centre in Sweden, serving a catchment area of approximately 2.8 million inhabitants, corresponding to approximately a quarter of the Swedish population. As the only designated regional trauma centre with neurosurgical and cardiothoracic surgical services, it receives all patients meeting national trauma team activation criteria based on physiological or anatomical parameters. A minority of patients are initially admitted to other regional hospitals and subsequently transferred.

The catchment area is predominantly urban, encompassing the Stockholm metropolitan region, with a smaller population residing on islands in the Stockholm archipelago. Road ambulance services cover nearly the entire region, with helicopter transport primarily used when transport times from peripheral areas can be reduced.

Upon arrival, trauma patients bypass the emergency department and are admitted directly to the trauma unit. Full trauma team activation mobilises a multidisciplinary team including trauma surgeons, anaesthesiologists, nurse anaesthetists, operating theatre nurses, orthopaedic surgeons and radiologists for initial assessment and management. The unit has immediate access to CT imaging, and hybrid operating theatres, enabling rapid transition to surgical or interventional radiological procedures when required.

The prehospital emergency services are supported by physician-staffed rapid response units covering the entire urban region and dispatched to acute life-threatening emergencies, including trauma, providing early advanced interventions.

In 2018, Karolinska University Hospital relocated to a new facility, with an immediate transition to the new trauma unit and no diversion of patients to other hospitals.

### Statistical analyses

To evaluate whether 30-day mortality changed over time while controlling for confounding variables, multivariable logistic regression was performed with 30-day mortality as the dependent variable and year of admission as a categorical predictor (reference year: 2017). The model was adjusted for age, sex, Injury Severity Score (ISS) category (≤ 15, 16–25, 26–35, 36–75), ASA score and injury type (penetrating/blunt). Results are presented as odds ratios (OR) with 95% confidence intervals (CI). Sensitivity analyses added Glasgow Coma Scale category and systolic arterial pressure on arrival, omitted ASA score, substituted the New Injury Severity Score for ISS, and were restricted to patients with ISS > 15 (Supplementary Table 2).

Time from arrival to computed tomography (CT) was summarised by year as median with interquartile range. Temporal changes were assessed using multivariable linear regression, with time to CT as the dependent variable and year as categorical predictor (reference year: 2017) The model was adjusted for age, sex, ISS category, ASA score, and injury type, and results are presented as adjusted mean differences with 95% CI. Given the right-skewed distribution of time to CT, a sensitivity analysis using quantile regression at the median was performed, yielding adjusted median differences with 95% CIs. The hospital relocation in October 2018 was examined separately by comparing time to CT before and after the move, adjusted for the same covariates as in the main time-to-CT model.

The median time to the first emergency procedure after arrival at the trauma unit was assessed for trauma patients from 2017 to 2022–2023. Emergency procedures included damage control thoracotomy or laparotomy, extraperitoneal pelvic packing, limb revascularisation, interventional radiology, craniotomy, and intracranial pressure device insertion. Procedures recorded in the registry as “other” were not classifiable into these categories and are not included in the analysis of emergency procedures. Each year was compared with 2017. Because emergency procedures are inherently immediate and less likely to be influenced by covariates, the unadjusted Mann–Whitney U test was used to evaluate differences in time to intervention.

Categorical variables are presented as proportions and percentages, and continuous variables as medians with interquartile ranges. A P value of < 0.05 was considered statistically significant. All statistical analyses were conducted using Stata/SE 19 (StataCorp, College Station, TX, USA).

### Sensitivity analyses

A sensitivity analysis was conducted restricting the cohort to patients with ISS > 15 (major trauma) to assess whether temporal changes in mortality and in time to CT were consistent in the most severely injured patients; for time to CT these analyses were performed using both linear and quantile regression. Further sensitivity analyses addressed the pooling of 2022 and 2023, as described above. Seasonal variation in case-mix was assessed by comparing calendar quarters with the chi-squared test. All sensitivity analyses are summarised in Supplementary Table 2.

## Results

Between January 2017 and August 2023, a total of 5,615 patients were recorded as full trauma team activation in the Karolinska Trauma Registry. Patient characteristics are summarised in Table 1. The median age was 41 years and approximately 75% were male. Blunt trauma dominated, with traffic accidents (31%) and falls (33%) as the most common injury mechanisms, followed by stab or gunshot wounds (24%).

**Table 1:** Characteristics of the study cohort

Count	5,615
**Age**,** median (IQR)**	41 (26–59)
**Male**,** count (%)**	4,125 (73.5)
**ASA score**,** count (%)** ASA 1 ASA 2 ASA 3 ASA 4	3,141 (56.1) 1,447 (25.8) 982 (17.5) 33 (0.6)
**Injury mechanism**,** count (%)** Traffic Motor vehicle accident Motorcycle accident Bicycle accident Pedestrian Traffic other Gunshot Stab wound Struck by blunt object Low energy fall High energy fall Blast injury Other	1,713 (30.6) 663 (11.8) 391 (7.0) 416 (7.4) 180 (3.2) 63 (1.1) 315 (5.6) 1,002 (17.9) 571 (10.2) 621 (11.1) 1,238 (22.1) 34 (0.6) 101 (1.8)
**Injury type**,** count (%)** Penetrating Blunt	1,324 (23.6) 4,291 (76.4)
**Injury intention**,** count (%)** Accident Self-inflicted Assault Other	3,723 (66.8) 580 (10.4) 1,254 (22.5) 14 (0.3)
**Care level at scene**,** count (%)** No field care Basic life support ALS – no physician ALS – physician field care Other or missing	83 (1.5) 1 (0.0) 3,613 (64.3) 1,421 (25.3) 497 (8.9)
**Transportation type**,** count (%)** Ground ambulance Helicopter ambulance Other or missing	3,899 (69.4) 1,136 (20.2) 580 (10.3)
**Intubated before hospital arrival**,** count (%)**	289 (5.1)
**GCS on arrival**,** count (%)** 14–15 9–13 3–8	4,076 (81.9) 589 (11.8) 309 (6.2)
**ISS**,** median (IQR)**	9 (2–18)
**ISS categories**,** count (%)** ≤ 15 16–25 26–35 36–75	3,808 (67.9) 949 (16.9) 519 (9.3) 332 (5.9)
**Serious injury by body region**,** count (%)** Head* Face* Neck* Thoracic* Abdominal* Spinal* Lower extremity* Upper extremity*	1,082 (19.6) 91 (1.6) 112 (2.0) 1,286 (23.3) 463 (8.4) 429 (7.8) 733 (13.3) 120 (2.2)
Shock on arrival**,** count (%)**	392 (7.1)
Massive transfusion***,** count (%)**	369 (7.0)
**Highest care level**,** count (%)** Emergency department General ward Operating theatre High dependency unit Intensive care unit	375 (6.7) 2,399 (42.7) 1,149 (20.5) 268 (4.8) 1,424 (25.4)
**Hospital length of stay**,** count (%)** 0–2 days 3–7 days >7 days	2,472 (44.0) 1,717 (30.6) 1,426 (25.4)
**GOS at discharge**,** count (%)** 1 2 3 4 5	438 (7.8) 10 (0.2) 1,558 (27.7) 1,851 (33.0) 1,758 (31.3)
**30-day mortality**,** count (%)**	483 (8.7)

Continuous data presented as median (IQR). Categorical data as n (%). *Maximum AIS ≥ 3 for the body region; patients without recorded injury coding for a region are excluded from the denominator. **SAP ≤ 90 mmHg. ***>10 units pRBC in 24 h or > 4 units in 1 h. AIS, Abbreviated Injury Scale; ALS, advanced life support; ASA, American Society of Anesthesiologists; GCS, Glasgow Coma Scale; GOS, Glasgow Outcome Scale; ISS, Injury Severity Score; SAP, systolic arterial pressure

Missing data: massive transfusion *n* = 366 (6.5%); GCS *n* = 641 (11.4%); SAP *n* = 101 (1.8%); injury intention *n* = 44 (0.8%); 30-day mortality *n* = 38 (0.7%); injury mechanism *n* = 20 (0.4%); ASA *n* = 12 (0.2%); ISS *n* = 7 (0.1%). All other variables in this table are complete except where stated above. Percentages use the number of patients with non-missing data for each variable as the denominator. For care level at scene and transportation type, patients not assignable to a defined category are reported in the table as “Other or missing” rather than as missing data

Regarding pre-hospital care, 70% of patients were transported by road ambulance and 20% by helicopter ambulance, while 25% received physician-provided advanced prehospital care. More than 30% of patients met criteria for major trauma (ISS > 15). The most common serious injuries (maximum AIS ≥ 3) involved the thorax (23%), head (20%), and lower extremities (13%).

**Fig. 1:**
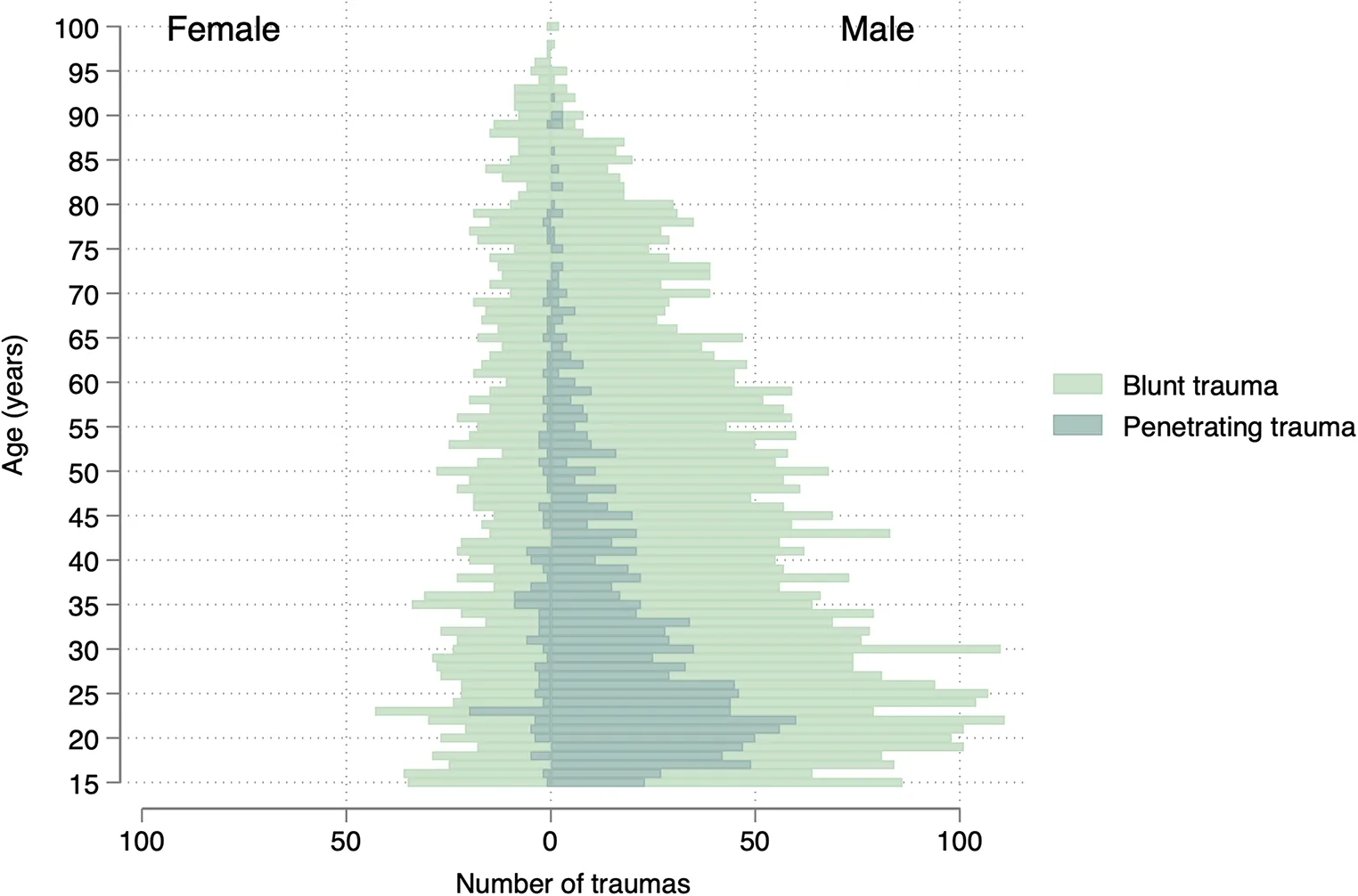
Number of trauma patients admitted during the study period divided by age, sex and penetrating/blunt trauma

Approximately 25% of patients were admitted to the ICU, and nearly half had a hospital stay of less than three days. At discharge, 30% of patients achieved a Glasgow Outcome Scale score of 5, indicating full recovery, and 30-day mortality was 8.7%. Most trauma patients were younger adults with less severe injuries (Supplementary Fig. 1), and penetrating injuries predominantly affected young men (Fig. 1).

**Fig. 2:**
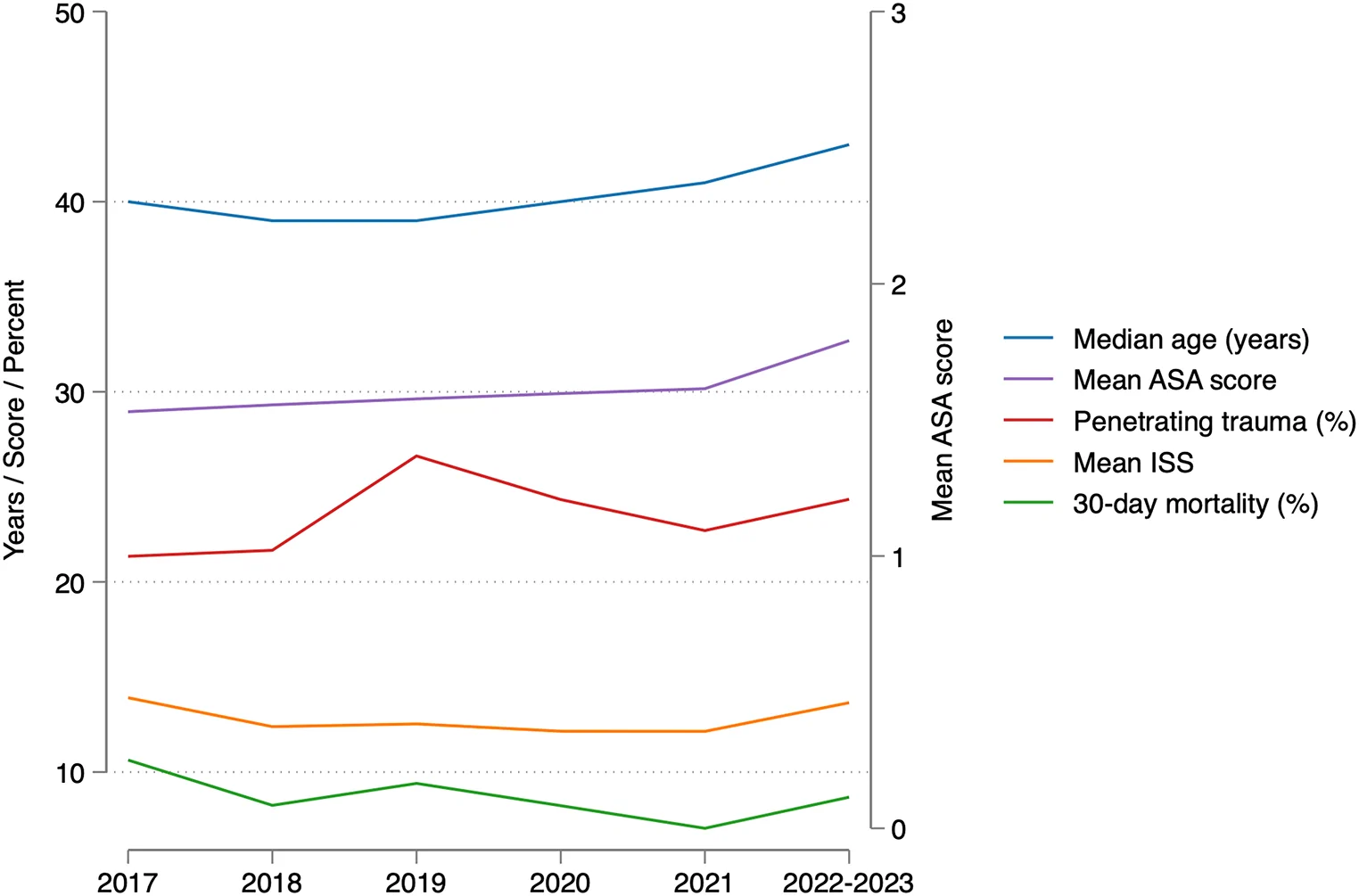
Median age (years, left y-axis), percent penetrating trauma (left y-axis), mean Injury Severity Score (left y-axis), percent 30-day mortality (left y-axis), mean ASA score (right y-axis). The x-axis represents each year during the study period. ASA, American Society of Anesthesiologists

Over the study period, patient age and ASA scores increased, while injury type distribution and ISS remained relatively unchanged (Supplementary Table 1). In contrast, 30-day mortality appeared to decline over time (Fig. 2).

Crude 30-day mortality ranged from 7.0% in 2021 to 10.6% in 2017, with intermediate values between 8.2% and 9.4% across other years. Among patients with ISS ≤ 15, 30-day mortality remained low throughout the study period, with overall mortality of 2.5%. In contrast, patients with ISS > 15 had substantially higher mortality, decreasing from 26.9% in 2017 to 17.4% in 2022–2023, yielding an overall mortality of 21.6%.

In multivariable logistic regression with 2017 as reference, adjusted for age, sex, ISS category, ASA score and injury type, the odds of 30-day mortality were progressively lower (Table 2) over the study period, reaching statistical significance in 2021 (OR 0.61, 95% CI 0.40–0.91, *P* = 0.016) and 2022–2023 (OR 0.61, 95% CI 0.43–0.88, *P* = 0.008). The model included 5,559 (99%) of the 5,615 patients.

**Table 2:** Multivariable logistic regression analysis of 30-day mortality: adjusted odds ratios with 2017 as reference year (adjusted for age, sex, ISS category, ASA score, and injury type)

	OR*	95% CI	*P*
**Year of admission**			
2017	1.00	Reference	
2018	0.80	0.54–1.21	0.293
2019	0.85	0.57–1.27	0.429
2020	0.78	0.53–1.15	0.214
2021	0.61	0.40–0.91	**0.016**
2022–2023	0.61	0.43–0.88	**0.008**

Year of admission as categorical predictor (reference: 2017)

*Adjusted for age, sex, ISS category (≤ 15, 16–25, 26–35, 36–75), ASA score and injury type (penetrating/blunt). The model included 5,559 of 5,615 patients; 56 were excluded because of missing values in the outcome or a covariate

OR, odds ratio; CI, confidence interval. Significant P-values (< 0.05) in bold

**Fig. 3:**
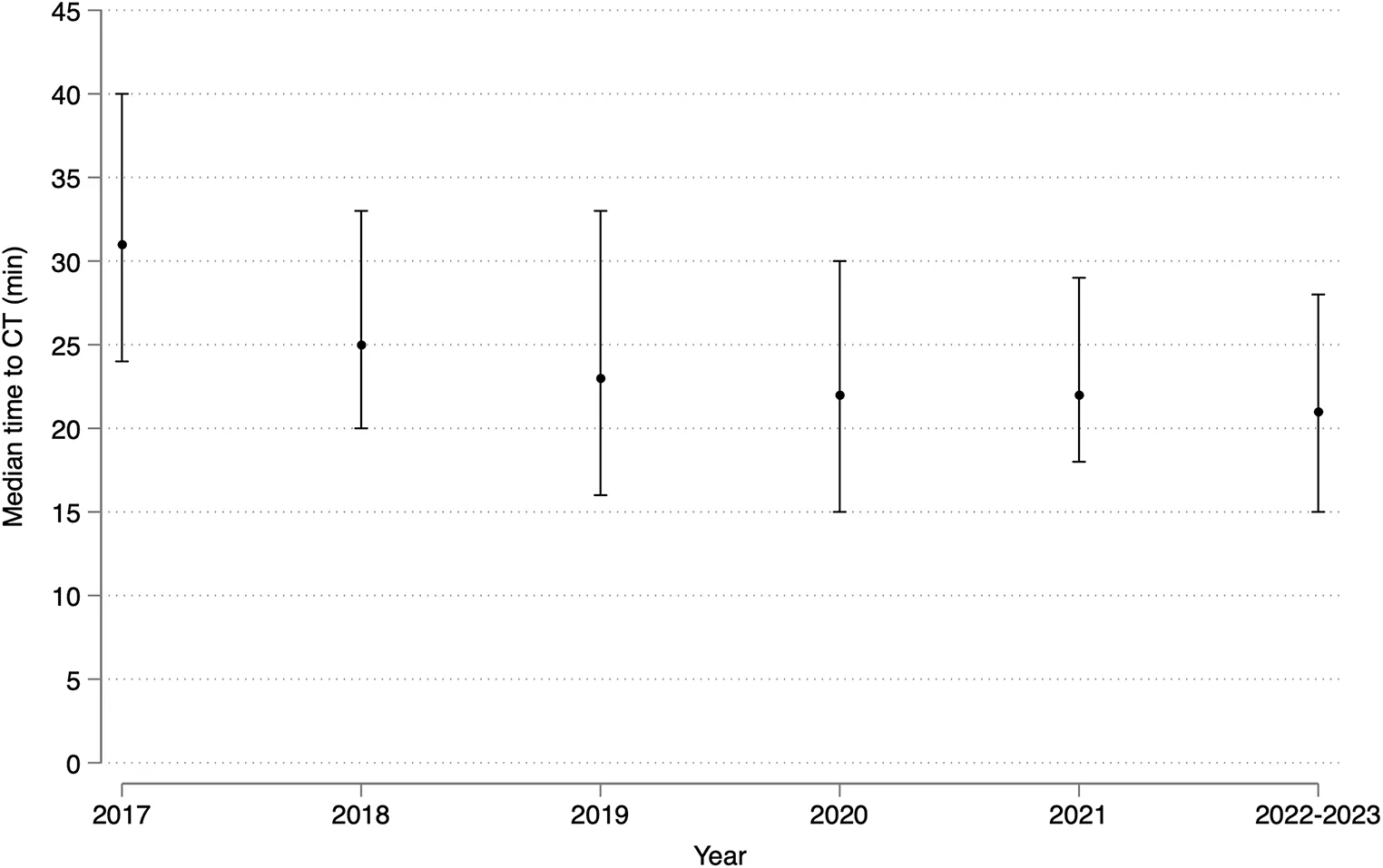
Median time (minutes) from arrival to the trauma unit until CT initiation, with interquartile range, y-axis. The x-axis represents each year during the study period. CT, computed tomography

The median time to CT after hospital arrival decreased from 31 min (IQR 24–40) in 2017 to 21 min (IQR 15–28) in 2022–2023 (Fig. 3). In adjusted linear regression analysis, time to CT was shorter in 2021 (− 6.6 min; 95% CI − 13.0 to − 0.3, *P* = 0.040) and in 2022–2023 (− 7.5 min; 95% CI − 13.5 to − 1.5, *P* = 0.014) compared with the reference year 2017, whereas no differences were observed for 2018–2020. Because the distribution of time to CT was right-skewed, the analysis was repeated using quantile regression, which gave a larger and more precisely estimated reduction in the median of 10.4 min in 2022–2023 (95% CI − 11.5 to − 9.2, *P* < 0.001). The model included 5,073 patients; four of the 5,077 patients with a recorded time to CT were excluded because of missing covariate data.

Median time to CT was 29 min (IQR 22–38) before the hospital relocation in October 2018 and 22 min (IQR 16–30) afterwards. The adjusted mean difference was − 4.5 min (95% CI − 8.5 to − 0.5) by linear regression, and the adjusted median difference was − 7.0 min (95% CI − 7.8 to − 6.2) by quantile regression.

Computed tomography was not performed in 538 patients (9.6%) and 112 patients (2.0%) underwent an emergency procedure without a preceding CT. These 112 patients were more severely injured and more often haemodynamically compromised than the remainder of the cohort (ISS > 15 in 58.9% versus 31.6%, systolic arterial pressure ≤ 90 mmHg in 40.0% versus 6.5%, and 30-day mortality 42.9% versus 8.0%).

The median time to the first emergency procedure was 105 min in 2017 and 87 min in 2022–2023 (Supplementary Fig. 2). However, this difference was not statistically significant (*P* = 0.45), indicating no significant reduction in time to surgery over the study period. The composition of emergency procedures changed over the study period: craniotomy decreased from 33.3% to 13.8% of procedures and damage control laparotomy increased from 22.2% to 42.5%, while the proportion undergoing interventional radiology was stable (11.1% and 12.5%) (Supplementary Table 3). Median time from arrival to craniotomy decreased from 128 min (IQR 101–268) in 2017 to 84 min (IQR 61–111) in 2022–2023, whereas median time to interventional radiology increased from 152 min (IQR 126–204) to 194 min (IQR 112–368). Both comparisons rest on small numbers, and no formal test was performed.

Physician field care increased from 5.8% of patients in 2017 to 20.4% in 2018 and 30.8% in 2022–2023. No changes were observed in transport or on-scene times (Supplementary Fig. 3).

### Sensitivity analyses

In analyses restricted to patients with ISS > 15, the estimated reduction in the odds of 30-day mortality was more pronounced than in the whole cohort. For time to CT in this subgroup, linear regression showed no difference in the mean. Quantile regression, however, showed an 11.4-minute reduction in the median in 2022–2023 compared with 2017, consistent with the unadjusted medians shown in Supplementary Fig. 4. The discrepancy may reflect the greater sensitivity of linear regression to extreme time-to-CT values.

When 2022 and 2023 were entered as separate categories, the reduction in mortality was similar in both years. Injury mechanism varied by season, with penetrating injuries more frequent in the first and fourth quarters. Injury severity showed no significant seasonal variation, and 30-day mortality ranged from 8.4% to 9.1% across quarters. Restricting all years to January-August reduced the cohort by 29%. In this analysis, the reduction in time to CT persisted, whereas the mortality estimate was attenuated and no longer statistically significant (OR 0.76, 95% CI 0,50-1.17) Model estimates are reported in Supplementary Table 2.

### Missing data

Missing data were limited for 30-day mortality and the covariates used in the primary models (< 1% per variable; Table 1). Complete-case analysis was used throughout. The mortality model included 5,559 of 5,615 patients, and the time-to-CT model included 5,073 patients. No imputation was performed.

## Discussion

This study explores changes in demographics, injury severity, trauma care and mortality at a major trauma centre in Sweden, covering the period from January 2017 to August 2023. Despite an aging trauma patient population with increasing comorbidities, a reduction in 30-day mortality was observed. Evaluating trauma system performance over time is inherently difficult, as concurrent changes in demographics, case-mix and clinical practice may confound observed outcomes. Additional findings include a reduction in time to CT over the study period, which was at least as large among severely injured patients, in whom median times were lower than those reported by many other centres [9]. Time to CT was used as an indicator of trauma system efficiency; although shorter time to CT and lower mortality were observed over the same period, the present study was not designed to assess whether these changes were causally related.

The increased presence of physicians at the scene and advanced pre-hospital interventions, including airway management and blood transfusions, may have contributed to these improvements, reflecting increased trauma system efficiency [10]. Intrahospital factors, such as regular trauma team training and continuous staff education, may also have contributed to the observed improvements in outcomes.

The Swedish trauma population resembles that of other European countries and North America, with traffic accidents, falls, and intentional injuries as the most common mechanisms of injury [11]. The cohort is predominantly male, with penetrating injuries more frequent among younger patients, consistent with patterns observed in other studies [12]. Most patients sustain mild injuries (ISS ≤ 15) and are young to middle-aged.

An emerging challenge in Sweden, as in several other European countries, is the increasing prevalence of illegal firearms and gun violence, reflected in the rise of gun-related homicides – the homicide rate in Sweden now exceeds that of many other countries [13]. Despite this, the proportion of penetrating injuries did not increase consistently over the study period, ranging between 21% and 27% without a clear direction, and gunshot wounds remained stable at around 5–6% of admissions, with no accompanying rise in mortality or injury severity suggestive of increased fatal violence. A previous study indicates that a substantial proportion of fatalities due to penetrating injuries occur in the prehospital setting, where patients are pronounced dead at the scene, and thus not captured in the Karolinska Trauma Registry [14]. This may explain why we did not observe an increase in severe penetrating traumas in our study.

Advanced age is a well-established risk factor for increased mortality following trauma [15, 16]. Older adults have a higher mortality risk following severe trauma, independent of Injury Severity Score (ISS) and an elevated long-term risk of death [17, 18]. This vulnerability reflects increased frailty, higher comorbidity, reduced physiological reserves and impaired ability to recover from traumatic injuries [19]. In addition, elderly trauma patients more frequently use chronic medications, including anticoagulants, increasing the risk of bleeding complications. The geriatric population has increased globally and in Sweden and is expected to continue rising [20]. Consistent with this demographic shift, trauma patients in our cohort became older over time, accompanied by increasing ASA scores indicating a more compromised population [21]. The pre-injury ASA score is a well-established independent predictor of both mortality and readmission following trauma [22, 23].

Comparing outcomes across trauma systems over time is challenging, as it is difficult to simultaneously control for changes in case-mix, an aging population, medical advancements, and regional variations. Although some trauma centres have reported reduction in mortality over time [24, 25], contemporary data remain limited. It is well-established, however, that severely injured patients have improved survival when treated at trauma centres compared with non-trauma centres, a finding also demonstrated in a Swedish context [26, 27].

In this study, we observed a reduction in the adjusted 30-day mortality. By employing multivariable logistic regression adjusted for relevant confounders, rather than relying on crude mortality rates, we aimed to provide a more robust assessment of changes in mortality over time. The direction of the association was consistent across model specifications, although the estimate was attenuated in the season-matched analysis (Supplementary Table 2). However, the specific factors driving the observed mortality reduction remain difficult to isolate, and we acknowledge that our analysis cannot establish causality.

Our findings are consistent with a recent Danish study demonstrating a reduction in 30-day mortality between 2010 and 2019, (adjusted OR per year 0.94, 95% CI 0.90–0.99), suggesting improved outcome despite increasing age and injury severity over time [28]. Although that study and an earlier comparison of Karolinska University Hospital and Oslo University Hospital – Ullevål (2009–2011) reported somewhat lower crude mortality, direct comparisons are challenging due to differences in case mix [28, 29]. Notably, our cohort included a higher proportion of patients with penetrating injury.

To further assess trauma system performance, we focused on variables that were measurable within our trauma registry data. Given the importance of timely interventions in trauma care, we analysed time from hospital arrival to CT. Rapid access to CT is essential for the prompt identification and assessment of injuries requiring urgent intervention, with evidence supporting its role in improving survival outcomes [30, 31], . Delays in obtaining CT imaging are known to adversely affect outcomes [32–34]. Although Advanced Trauma Life Support guidelines recommend emergency surgery before CT in haemodynamically unstable trauma patients, the majority of trauma patients undergo CT shortly after hospital arrival [35]. Shorter time to CT has been associated with reduced hospital length of stay, suggesting it may serve as an indirect measure of trauma system efficiency [36]. The NHS Standard Contract for Major Trauma Services specifies a requirement for CT within 60 min of arrival, ideally within 30 min [37]. Reported times to CT vary considerably, with mean times of 87 min in severe traumatic brain injury [38], and median times of 60–82 min in polytrauma patients [9, 39, 40].

The median time to CT of 21 min in 2022–2023 is substantially shorter than that reported in many previous studies. However, direct comparisons between trauma centres are challenging due to differences in patient characteristics, trauma volumes and injury patterns. This finding also applies when analyses are restricted to severely injured patients. The short time to CT may reflect direct admission to the trauma unit with immediate access to a dedicated multidisciplinary team and on-site CT, bypassing the emergency department. The hospital relocation in October 2018 coincided with shorter time to CT, but any contribution of the move cannot be distinguished from the underlying temporal trend.

Shorter time to CT should not be regarded as a target for all patients. In haemodynamically unstable patients with suspected bleeding who do not respond to resuscitation, CT must not delay haemorrhage control. The observed reduction in time to CT may indicate improved system efficiency in patients for whom imaging is appropriate.

Short time to emergency procedures is likely important for survival, and a median time of approximately 90 min is consistent with previous reports [41, 42]. Interpretation is challenging, as many emergency procedures may not ultimately be time-critical, and the registry lacks data on the true time-sensitivity of these procedures. Median time to craniotomy shortened over the period, whereas time to interventional radiology did not. Nonetheless, ongoing efforts to further reduce time to surgery may contribute to improved outcomes.

Another factor sometimes proposed as contributing to improved outcomes is the increased physician presence at the scene of trauma. Although transport times remained unchanged, the proportion of patients receiving advanced pre-hospital care increased. While specific interventions could not be assessed, advanced airway management, thoracostomy, haemorrhage-control interventions, and pre-hospital blood transfusion may have contributed to improved outcomes [43, 44]. Additionally, the presence of experienced physicians at scene likely improves prioritisation and decision-making, including appropriate destination determination and decisions regarding initiation or withholding of specific treatments [45, 46]. Improved airway management is particularly relevant as physician-performed intubation has been associated with higher first-pass success rates [47].

### Strengths and limitations

This study has several limitations. As a single-centre study, generalisability to other settings may be limited. The retrospective analysis of prospectively collected data remains susceptible to residual confounding. Study inclusion was restricted to patients aged > 15 years admitted following full trauma team activation. Patients managed following limited or no trauma team activation were therefore not represented, potentially introducing selection bias. Because prehospital deaths are not registered, survivor bias is also possible, and changes in prehospital care may have altered the composition of the admitted patients in ways we cannot quantify. Changes in coding or registry completeness may have introduced information bias, as suggested by temporal shifts in ASA classification and a relatively high proportion of missing massive transfusion data in 2017; however, omitting ASA score from the mortality model did not materially alter the result. The study period included the COVID-19 restrictions in 2020, although registry data showed no reduction in trauma admissions or change in injury mechanisms. Unequal calendar-month coverage resulting from pooling 2022 with the first eight months of 2023 was addressed in a season-matched sensitivity analysis.

Strengths include a robust trauma registry with minimal missing data for the variables used in the primary analyses and little loss to follow-up. Although this was a single-centre study, Karolinska University Hospital has a large catchment area and manages approximately 17% of all trauma cases in Sweden, supporting the relevance of the findings to Swedish trauma care.

## Conclusion

Our findings suggest a reduction in risk-adjusted 30-day mortality and shorter median time to CT over the study period, with the latter at least as marked among severely injured patients. Although the specific drivers of these changes are difficult to isolate in this observational study, the results are consistent with improvements within the trauma system. These findings should be interpreted as parallel indicators of trauma system performance, not as evidence of a causal relationship between time to CT and mortality.

## Supplementary Information

Below is the link to the electronic supplementary material.

Supplementary Material 1

## Data Availability

The data that support the findings of this study are available from Trauma Registry Karolinska, but restrictions apply to the availability of these data and are not publicly available. Data are however available from the authors upon reasonable request and with permission of Swedish Ethical Review Authority.

## Abbreviations

ASA: American society of anesthesiologists
AIS: Abbreviated injury scale
CI: Confidence intervals
CT: Computed tomography
GCS: Glasgow coma scale
ICU: Intensive care unit
ISS: Injury severity score
LOS: Length of stay at hospital
SAP: Systolic arterial blood pressure
