# Supplementary material for: Trauma care performance and patient outcomes - insights from a major trauma centre in Sweden
Source: Eur J Trauma Emerg Surg. 2026 Oct 27;52(1):334. doi: 10.1007/s00068-026-03364-5 (PMC13714035; doi:10.1007/s00068-026-03364-5)
Supplement: Supplementary file 1 [file 68_2026_3364_MOESM1_ESM.docx]

**Supplementary Materials**

*Trauma care performance and patient outcomes – insights from a major trauma centre in Sweden*

**Supplementary Table 1**

*Patient characteristics by year of admission*

Patient demographics, injury characteristics, injury mechanism, prehospital care and outcomes stratified by year of admission (2017–2023, with 2022 and 2023 combined). Continuous data are presented as median (interquartile range) and categorical data as n (%). Percentages use the number of patients with non-missing data for each variable as the denominator, so denominators differ between rows. Massive transfusion was not recorded for 202 patients in 2017 (24.8%) compared with 0.1–8.7% in later years, and the apparent increase over time should be interpreted accordingly. File format: .docx

|  | **2017** | **2018** | **2019** | **2020** | **2021** | **2022–2023** |
| --- | --- | --- | --- | --- | --- | --- |
|  | **N=815** | **N=817** | **N=815** | **N=937** | **N=925** | **N=1,306** |
| **Female sex** | 212 (26.0) | 217 (26.6) | 207 (25.4) | 238 (25.4) | 233 (25.2) | 383 (29.3) |
| **Age, median (IQR)** | 40 (26–57) | 39 (27–57) | 39 (25–59) | 40 (26–58) | 41 (27–59) | 43 (27–61) |
| **ISS, median (IQR)** | 10 (4–19) | 9 (2–17) | 9 (2–17) | 9 (1–17) | 9 (1–17) | 10 (2–20) |
| **ASA score** |  |  |  |  |  |  |
| 1 | 490 (60.3) | 496 (60.8) | 486 (60.1) | 554 (59.3) | 530 (57.4) | 585 (44.8) |
| 2 | 218 (26.8) | 191 (23.4) | 182 (22.5) | 208 (22.2) | 224 (24.2) | 424 (32.5) |
| 3 | 102 (12.5) | 125 (15.3) | 138 (17.1) | 169 (18.1) | 166 (18.0) | 282 (21.6) |
| 4 | 3 (0.4) | 4 (0.5) | 3 (0.4) | 4 (0.4) | 4 (0.4) | 15 (1.1) |
| **ISS category** |  |  |  |  |  |  |
| 0–15 | 539 (66.3) | 560 (68.5) | 562 (69.0) | 669 (71.4) | 646 (69.8) | 832 (64.0) |
| 16–25 | 139 (17.1) | 142 (17.4) | 126 (15.5) | 136 (14.5) | 151 (16.3) | 255 (19.6) |
| 26–35 | 73 (9.0) | 69 (8.4) | 81 (9.9) | 84 (9.0) | 80 (8.6) | 132 (10.1) |
| 36–75 | 62 (7.6) | 46 (5.6) | 46 (5.6) | 48 (5.1) | 48 (5.2) | 82 (6.3) |
| **Blunt trauma**  **Penetrating trauma** | 641 (78.7)  174 (21.3) | 640 (78.3)  177 (21.7) | 598 (73.4)  217 (26.6) | 709 (75.7)  228 (24.3) | 715 (77.3)  210 (22.7) | 988 (75.7)  318 (24.3) |
| **Injury mechanism** |  |  |  |  |  |  |
| **Motor vehicle accident** | 128 (15.7) | 104 (12.8) | 90 (11.1) | 106 (11.4) | 101 (10.9) | 134 (10.3) |
| **Motorcycle accident** | 71 (8.7) | 57 (7.0) | 45 (5.6) | 69 (7.4) | 65 (7.0) | 84 (6.4) |
| **Bicycle accident** | 40 (4.9) | 57 (7.0) | 58 (7.2) | 76 (8.1) | 77 (8.3) | 108 (8.3) |
| **Pedestrian** | 27 (3.3) | 33 (4.1) | 36 (4.4) | 29 (3.1) | 19 (2.1) | 36 (2.8) |
| **Traffic, other** | 13 (1.6) | 11 (1.4) | 8 (1.0) | 7 (0.8) | 10 (1.1) | 14 (1.1) |
| **Gunshot** | 45 (5.5) | 48 (5.9) | 47 (5.8) | 60 (6.4) | 43 (4.7) | 72 (5.5) |
| **Stab wound** | 123 (15.1) | 128 (15.8) | 169 (20.9) | 169 (18.1) | 166 (18.0) | 247 (19.0) |
| **Struck by blunt object** | 78 (9.6) | 73 (9.0) | 75 (9.3) | 109 (11.7) | 104 (11.3) | 132 (10.1) |
| **Low energy fall** | 79 (9.7) | 74 (9.1) | 82 (10.1) | 97 (10.4) | 121 (13.1) | 168 (12.9) |
| **High energy fall** | 188 (23.1) | 208 (25.6) | 183 (22.6) | 184 (19.7) | 203 (22.0) | 272 (20.9) |
| **Blast injury** | 6 (0.7) | 4 (0.5) | 3 (0.4) | 5 (0.5) | 3 (0.3) | 13 (1.0) |
| **Other** | 15 (1.8) | 15 (1.8) | 14 (1.7) | 22 (2.4) | 12 (1.3) | 23 (1.8) |
| **GCS on arrival** |  |  |  |  |  |  |
| 14–15 | 569 (80.5) | 588 (81.9) | 588 (83.8) | 702 (83.3) | 712 (84.2) | 917 (79.2) |
| 9–13 | 88 (12.4) | 85 (11.8) | 73 (10.4) | 96 (11.4) | 91 (10.8) | 156 (13.5) |
| 3–8 | 50 (7.1) | 45 (6.3) | 41 (5.8) | 45 (5.3) | 43 (5.1) | 85 (7.3) |
| **SAP >90 mmHg** | 741 (93.0) | 754 (93.8) | 729 (91.7) | 861 (92.6) | 855 (93.8) | 1,182 (92.6) |
| **Prehospital intubation** | 48 (5.9) | 49 (6.0) | 65 (8.0) | 40 (4.3) | 29 (3.1) | 58 (4.4) |
| **Physician field care** | 47 (5.8) | 167 (20.4) | 297 (36.4) | 264 (28.2) | 244 (26.4) | 402 (30.8) |
| **Massive transfusion** | 27 (4.4) | 40 (5.3) | 66 (8.9) | 68 (7.3) | 62 (6.7) | 106 (8.3) |
| **Time to CT, min, median (IQR)** | 31 (24–40) | 25 (20–33) | 23 (16–33) | 22 (15–30) | 22 (18–29) | 21 (15–28) |
| **30-day mortality** | 86 (10.6) | 67 (8.3) | 76 (9.4) | 77 (8.2) | 65 (7.0) | 112 (8.7) |

Supplementary Table 2

Sensitivity analyses for 30-day mortality and time to computed tomography

Odds ratios for 30-day mortality and differences in minutes for time to computed tomography, each comparing 2022–2023 with 2017, under alternative model specifications: addition of Glasgow Coma Scale category and systolic arterial pressure, omission of ASA score, substitution of the New Injury Severity Score for ISS, restriction to patients with ISS >15, restriction of every year to January–August, and entry of 2022 and 2023 as separate categories. Time to CT is analysed by both linear and quantile regression. File format: .docx

| **Analysis** | **Estimate** | **95% CI** | **P** | **n** |
| --- | --- | --- | --- | --- |
| **30-day mortality, odds ratio for 2022–2023 vs 2017** |  |  |  |  |
| Primary model | 0.61 | 0.43 to 0.88 | 0.008 | 5,559 |
| Adding GCS and systolic pressure | 0.57 | 0.31 to 1.03 | 0.062 | 4,879 |
| Primary model, same patients as above | 0.54 | 0.31 to 0.93 | 0.028 | 4,879 |
| Excluding ASA score | 0.65 | 0.45 to 0.92 | 0.016 | 5,570 |
| NISS instead of ISS | 0.69 | 0.48 to 0.99 | 0.046 | 5,559 |
| Restricted to ISS > 15 | 0.51 | 0.35 to 0.75 | <0.001 | 1,768 |
| January–August of every year | 0.76 | 0.50 to 1.17 | 0.209 | 3,933 |
| 2022 analysed separately | 0.61 | 0.40 to 0.91 | 0.016 | 5,559 |
| 2023 (January–August) analysed separately | 0.62 | 0.39 to 0.98 | 0.040 | 5,559 |
| **Time to CT, minutes, 2022–2023 vs 2017** |  |  |  |  |
| Linear regression (mean) | -7.5 | -13.5 to -1.5 | 0.014 | 5,073 |
| Quantile regression (median) | -10.4 | -11.5 to -9.2 | <0.001 | 5,073 |
| ISS > 15, linear regression (mean) | 0.1 | -15.3 to 15.5 | 0.986 | 1,537 |
| ISS > 15, quantile regression (median) | -11.4 | -14.0 to -8.8 | <0.001 | 1,537 |
| January–August of every year | -7.3 | -14.1 to -0.4 | 0.038 | 3,587 |

Supplementary Table 3

Emergency procedures by year of admission

Number and proportion of patients undergoing each category of emergency procedure, and median time in minutes from arrival at the trauma unit to the procedure, for each year during the study period. Patients recorded as having no emergency intervention, another intervention, or an unknown intervention are excluded. A total of 1,262 patients underwent a procedure recorded as “other” and are not included in this table; 3,753 patients had no emergency intervention. File format: .docx

|  | **2017** | **2018** | **2019** | **2020** | **2021** | **2022–2023** |
| --- | --- | --- | --- | --- | --- | --- |
| **Patients undergoing an emergency procedure** | **n = 72** | **n = 89** | **n = 100** | **n = 87** | **n = 91** | **n = 160** |
| **Procedure, n (% of procedures)** |  |  |  |  |  |  |
| Damage control thoracotomy | 10 (13.9) | 11 (12.4) | 16 (16.0) | 10 (11.5) | 9 (9.9) | 13 (8.1) |
| Damage control laparotomy | 16 (22.2) | 23 (25.8) | 38 (38.0) | 43 (49.4) | 35 (38.5) | 68 (42.5) |
| Extraperitoneal pelvic packing | 0 (0.0) | 2 (2.2) | 0 (0.0) | 2 (2.3) | 0 (0.0) | 0 (0.0) |
| Limb revascularisation | 4 (5.6) | 5 (5.6) | 8 (8.0) | 5 (5.7) | 6 (6.6) | 11 (6.9) |
| Interventional radiology | 8 (11.1) | 10 (11.2) | 12 (12.0) | 10 (11.5) | 9 (9.9) | 20 (12.5) |
| Craniotomy | 24 (33.3) | 23 (25.8) | 14 (14.0) | 11 (12.6) | 16 (17.6) | 22 (13.8) |
| Intracranial pressure device insertion | 10 (13.9) | 15 (16.9) | 12 (12.0) | 6 (6.9) | 16 (17.6) | 26 (16.2) |
| **Time from arrival to procedure, min, median (IQR)** |  |  |  |  |  |  |
| All emergency procedures | 105 (68–158) | 101 (50–131) | 91 (50–128) | 85 (55–135) | 94 (60–161) | 87 (58–153) |
| Damage control laparotomy | 70 (20–98) | 54 (31–102) | 83 (52–121) | 75 (50–100) | 72 (47–102) | 74 (42–98) |
| Interventional radiology | 152 (126–204) | 136 (102–176) | 104 (52–152) | 146 (82–182) | 131 (109–800) | 194 (112–368) |
| Craniotomy | 128 (101–268) | 110 (87–243) | 121 (98–145) | 84 (77–90) | 122 (99–158) | 84 (61–111) |

**Supplementary Figure 1**

*Number of trauma patients by age and Injury Severity Score*

Stacked area graph showing the number of trauma patients admitted during the study period (y-axis) over different ages (x-axis), divided by Injury Severity Score categories (ISS 0–15, 16–25, 26–35, 36–75). File format: .tif

**Supplementary Figure 2**

*Median time from arrival to the trauma unit until first emergency procedure*

Median time in minutes (y-axis) from arrival at the trauma unit until the first emergency procedure (damage control thoracotomy or laparotomy, extraperitoneal pelvic packing, limb revascularisation, interventional radiology, craniotomy, or intracranial pressure device insertion) for each year during the study period (x-axis). File format: .tif

**Supplementary Figure 3**

*Prehospital times and physician field care*

Median time from alarm to arrival at the trauma unit (minutes), median time from arrival at the trauma scene to arrival at the trauma unit (minutes), median time from arrival at the trauma scene to departure from the trauma scene (minutes), and proportion of patients receiving physician field care (percent) for each year during the study period. File format: .tif

**Supplementary Figure 4**

*Median time from arrival to the trauma unit until CT for patients with ISS >15*

Median time in minutes (y-axis) from arrival at the trauma unit until CT initiation for patients with Injury Severity Score >15 (major trauma), with interquartile range. The x-axis represents each year during the study period. This sensitivity analysis was performed to assess whether temporal changes in time to CT were consistent in the most severely injured patients. Because the distribution of time to CT is right-skewed, the corresponding adjusted analysis was performed using quantile regression (Supplementary Table 2). File format: .tif
